# Identification of key genes related to immune infiltration in cirrhosis via bioinformatics analysis

**DOI:** 10.1038/s41598-022-26794-8

**Published:** 2023-02-01

**Authors:** Tong-Yue Du, Ya-Xian Gao, Yi-Shan Zheng

**Affiliations:** Department of Critical Care Medicine, The Second Hospital of Nanjing, Nanjing University of Chinese Medicine, No.-1-1, Zhongfu Road, Nanjing, 210003 China

**Keywords:** Predictive markers, Inflammation, Gene expression analysis, Biophysics, Immunology, Biomarkers, Diseases

## Abstract

Cirrhosis is the most common subclass of liver disease worldwide and correlated to immune infiltration. However, the immune-related molecular mechanism underlying cirrhosis remains obscure. Two gene expression profiles GSE89377 and GSE139602 were investigated to identify differentially expressed genes (DEGs) related to cirrhosis. Enrichment analysis for DEGs was conducted. Next, the immune infiltration of DEGs was evaluated using CIBERSORT algorithm. The hub DEGs with tight connectivity were identified using the String and Cytoscape databases, and the expression difference of these hub genes between normal liver and cirrhosis samples was determined. Moreover, in order to evaluate the discriminatory ability of hub genes and obtained the area under the receiver operating characteristic curve values in the GSE89377 and GSE139602 datasets. Finally, the association between hub DEGs and immune cell infiltration was explored by Spearman method. Among the 299 DEGs attained, 136 were up-regulated and 163 were down-regulated. Then the enrichment function analysis of DEGs and CIBERSORT algorithm showed significant enrichment in immune and inflammatory responses. And four hub DEGs (*ACTB, TAGLN, VIM, SOX9*) were identified, which also showed a diagnostic value in the GSE89377 and GSE 139,602 datasets. Finally, the immune infiltration analysis indicated that, these hub DEGs were highly related to immune cells. This study revealed key DEGs involved in inflammatory immune responses of cirrhosis, which could be used as biomarkers for diagnosis or therapeutic targets of cirrhosis.

## Introduction

Cirrhosis with an annual incidence of 15.33–132.6 per 100,000 people, is responsible for approximately 1.03 million deaths each year worldwide^1–3^. Multiple mechanisms are involved in cirrhosis, including immune infiltration, necroinflammation and fibrogenesis^4^. Currently, few studies have focused on the screening of the key genes in cirrhosis immunity infiltration, and the complicated mechanisms and signaling pathways involving the recruitment and activation of immunity cells remain unclear. In addition, previous studies related to cirrhosis immunity infiltration generally target at Kupffer cells and macrophages^5,6^. Therefore, identification of key genes of immune infiltration in cirrhosis has become a hotspot in liver disease research.

Microarray and bioinformatics analysis has been extensively used to screen genetic variations at the genome sequencing^7,8^. A recent bioinformatics study on the key genes in the transformation of liver cirrhosis into hepatocellular carcinoma has confirmed the significance of this method for the screening of liver disease genes^9^. In our study, GSE89377^10,11^ and GSE139602^12^, two gene expression profiles derived from Gene Expression Omnibus (GEO), were analyzed. Then, functional enrichment analysis of the DEGs was performed based on Gene Ontology (GO) and the Kyoto Encyclopedia of Genes and Genomes (KEGG) database, and hub DEGs with a high connectivity were detected. Subsequently, the infiltration condition of immune cells in cirrhosis was analyzed via CIBERSORT algorithm^13^. Finally, the correlation between hub DEGs and immune cells was revealed.

## Materials and methods

### Microarray data

The expression profiles of genes related to cirrhosis were acquired from the GEO (https://www.ncbi.nlm.nih.gov/geo/)^14^. Two gene expression profiles were acquired from human liver samples. Only normal liver and cirrhosis samples were selected in each dataset. The GSE89377 series contained 13 normal human liver tissue specimens and 12 human cirrhosis tissue specimens based on the GPL16947 platform (Illumina HumanHT-12 V3.0 expression biochip). The GSE139602 series contained 6 normal human liver tissue specimens and 20 human cirrhosis tissue specimens based on the GPL13667 platform ([HG-U219] Affymetrix Human Genome U219 array). The basic clinical information is presented in Supplement Table 1. Depending to the annotation information of platform, probes were transformed into corresponding gene symbols.

### Identification of DEGs between normal liver and cirrhosis

GEO2R, an interactive network instrument datasets in the GEO series (https://www.ncbi.nlm.nih.gov/geo/geo2r/)^15^, was used to identify DEGs between normal liver and cirrhosis samples. Genes without corresponding gene symbol, and genes with multiple probes were separately omitted. |Log_2_FC|> 0.5 and adjusted *P* < 0.05 were the threshold criteria for statistical significance. In order to detect the overlapping DEGs from the two datasets, the Venn map was applied with ‘VennDiagram’ package (version: 1.71) in the R version: 4.1.0 (http://www.R-project.org)^16^. Meanwhile, heatmap and volcano plot of cirrhosis-related DEGs were created by the ‘Complex Heatmaps’ (version: 2.12.0)^17^ and ‘ggplot2’ packages (version: 3.3.5)^18^.

### Functional enrichment analysies of the cirrhosis-related DEGs

To understand the biological function of the overlapping DEGs in cellular components (CCs), molecular functions (MFs), and biological processes (BPs), the ‘clusterProfiler’ package (version 4.0.5) was used to perform Gene Ontology (GO) and Kyoto Encyclopedia of Genes and Genome (KEGG) pathway enrichment analysis^19^.

### Immune cell infiltration of cirrhosis

CIBERSORT was applied on preprocessed gene expression profiles to speculate the cell composition of complex tissues. The LM22 gene file was used to define the 22 immune cell subcategories and analyze cirrhosis data, which were attained from the CIBERSORT web portal (https://www.nature.com/articles/nmeth.3337#MOESM207). CIBERSORT algorithm in R was using to assess immune cell infiltration of cirrhosis^20^. Then, an immune cell infiltration matrix with 22 types of immune cells proportions was obtained. The ‘ggpubr’ (version: 0.4.0) and ‘ComplexHeatmap’ packages (version: 2.8.0) were used to display the proportion of the 22 types of immune cells in all samples and the expression difference in the 22 types of immune cells in normal and cirrhosis samples, respectively. To compare immune cell differences between normal and cirrhosis samples, the ‘ggplot2’ package (version: 3.3.5) was applied to perform a batch statistical t-test and create a boxplot^21^.

### Construction of protein and protein interaction (PPI) network and identification of hub DEGs

PPI was constructed to identify the key DEGs and gene modules in cirrhosis. Briefly, the cirrhosis-related DEGs were imported into the STRING (http://string-db.org Version:11.5)^22^ online analysis software to predict the interaction between the proteins encoded by these genes with a medium confidence score of > 0.4. Next, based on the STRING analysis, a PPI network of these genes were constructed by the Cytoscape software platform (version: 3.7.1)^23^, and the top-10 related hub-DEGs were screened by Cytoscape plug-in software ‘cytoHubba’ based on mixed character calculation including EPC, Degree, MNC, MCC and MCODE alogorithm. In addition, according to genes screening by the above four methods, the top 4 hub-DEGs were identified using the OmicShare online tool (https://www.omicstudio.cn/tool/43) and the UpSet plot was obtained.

### Diagnostic value of hub DEGs as biomarkers in cirrhosis

ROC curve from ‘pROC’ package (version: 1.18.0)^24^ was used to test the sensitivity and specificity of the identified biomarkers in GSE89377 and GSE139602 datasets. The area under the ROC curve (AUC) value was utilized to determine the diagnostic effectiveness in discriminating cirrhosis from control samples.

### Correlation analyses between hub DEGs and infiltrating immune cells

The potential relations between the hub DEGs and infiltrating immune cells were explored by the Spearman correlation analysis in R, and the results was visualized using the ‘ggpubr’ package.

### Expression of hub genes in normal liver or cirrhosis sample

The expression analysis of hub genes between normal liver or cirrhosis samples from GSE89377 and GSE139602 was conducted by Student’s t-test. The ‘ggpubr’ package was used to create the box plots of the expression of hub genes.

## Results

### Identification of DEGs between normal liver and cirrhosis tissues

**Figure 1:**
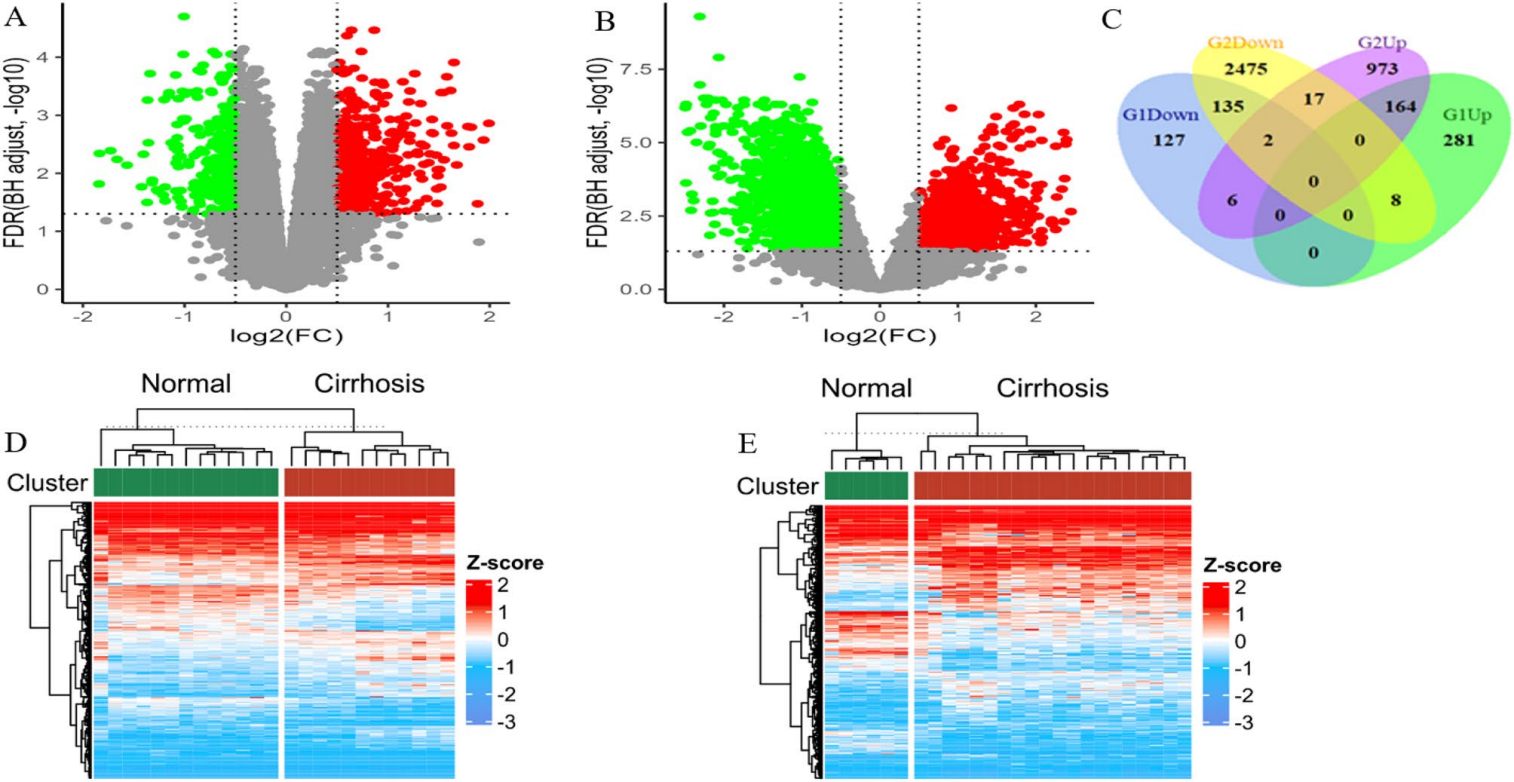
Volcano plots of all up- regulated genes (**A**) and down-regulated genes (**B**) show the DEGs between normal liver and cirrhosis samples from GSE89377 and GSE39602. Red indicates; greater expression and green indicates less expression. Venn Diagram (**C**) showing the upregulated and downregulated genes in the two datasets. The intersection of G1Down and G2Down represents downregulated genes in both datasets. The intersection of G1Up and G2Up represents upregulated genes in both datasets. An absolute log2 FC > 0.5 and an adjusted *P* value of < 0.05 cutoff was used to define differentially expressed mRNAs in cirrhosis. Heatmap (**D**) shows the genes expression changes between normal liver and cirrhosis samples from GSE89377. Heatmap (**E**) shows the genes expression change between normal liver and cirrhosis samples from GSE39602. DEGs: differentially expressed genes; G1Up: upregulated genes in GSE89377; G2Up: upregulated genes in GSE39602; G1Down: downregulated genes in GSE89377; G2Down: downregulated genes in GSE39602.

In GSE89377, a total of 723 cirrhosis-related DEGs were confirmed in the cirrhosis tissue, including 270 down-regulated genes and 453 up-regulated genes (Fig. 1A). In GSE139602, 3698 cirrhosis-related DEGs were confirmed in cirrhosis tissue compared with healthy liver tissues, including 2573 down-regulated genes and 1125 up-regulated genes (Fig. 1B).

### Screening the cirrhosis-related DEGs

A total of 299 cirrhosis-related genes were screen out among the overlapping DEGs from GSE89377 and GSE139602 in Supplement Table 2**.** The co-expression of DEGs were displayed using a Venn diagram (Fig. 1C)**.** The heat maps of the overlapping DEG expression in two samples are shown respectively (Fig. 1D, E).

### Functional enrichment analyses

**Figure 2:**
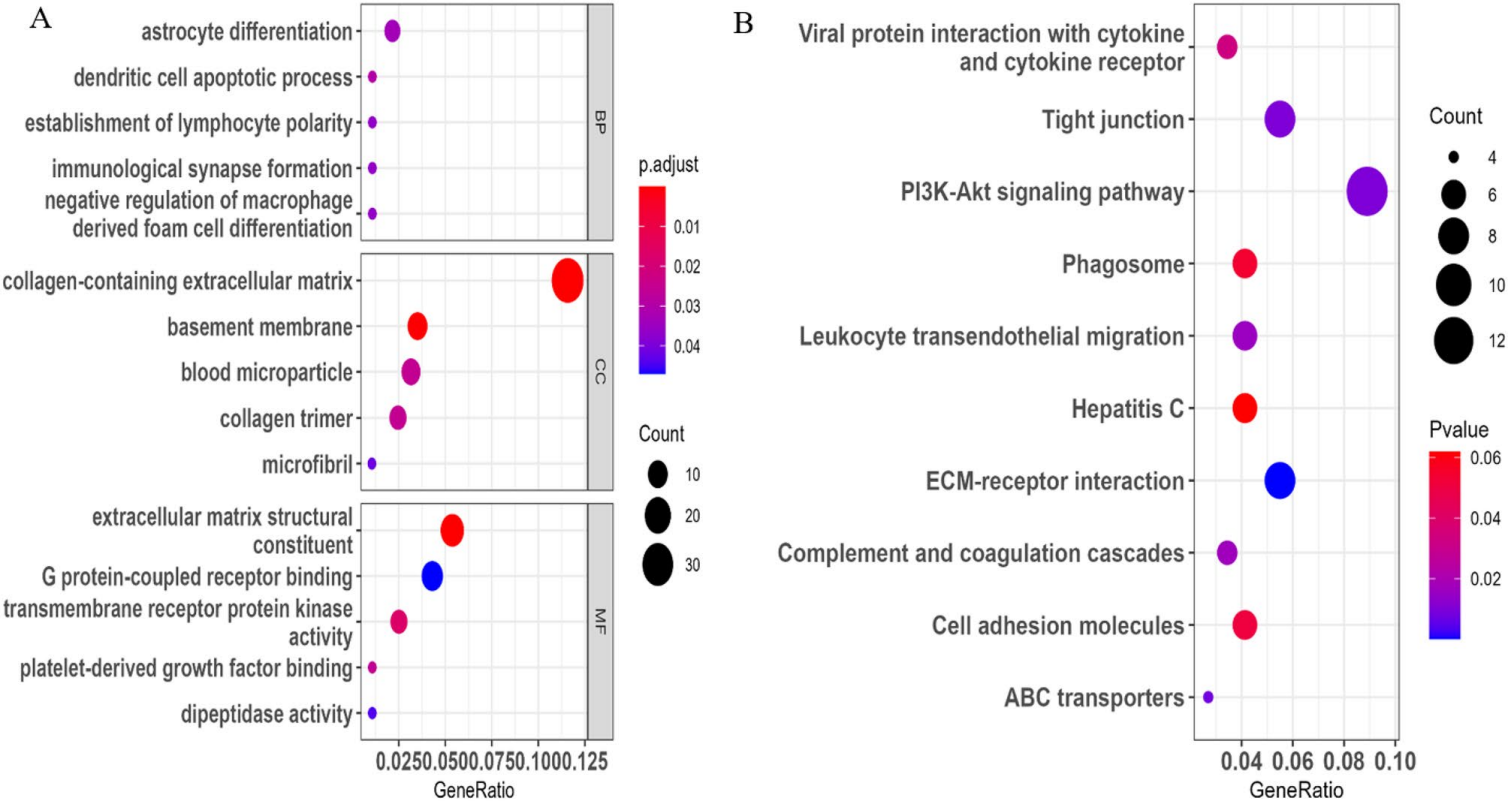
Enrichment analysis of cirrhosis-related DEGs. GO analysis (**A**) and KEGG pathway enrichment analysis (**B**). GO: Gene Ontology; KEGG: Kyoto Encyclopedia of Genes and Genomes; DEGs: differentially expressed genes.

The enriched biological processes (BPs) included astrocyte differentiation, dendritic cell apoptotic process, establishment of lymphocyte polarity, immunological synapse formation and negative regulation of macrophage derived foam cell differentiation. The cellular components (CCs) were primarily enriched in collagen-containing extracellular matrix, basement membrane, blood microparticle, collagen trimer and microfibril. The enriched molecular functions (MFs) included in external matrix structural constituent, G protein-coupled receptor binding, transmembrane receptor protein kinase activity, platelet-derived growth factor binding, and dipeptidase activity (Fig. 2A). KEGG pathway analysis displayed that viral protein interaction with cytokine and cytokine receptor, tight junction, cell adhesion molecules, leukocyte transendothelial migration, PI3K-Akt signaling pathway, phagosome, hepatitis C, ECM-receptor interaction, complement and coagulation cascades, and ABC transporters were enriched (Fig. 2B).

### Composition of infiltrating immune cells between normal liver and cirrhosis tissues

**Figure 3:**
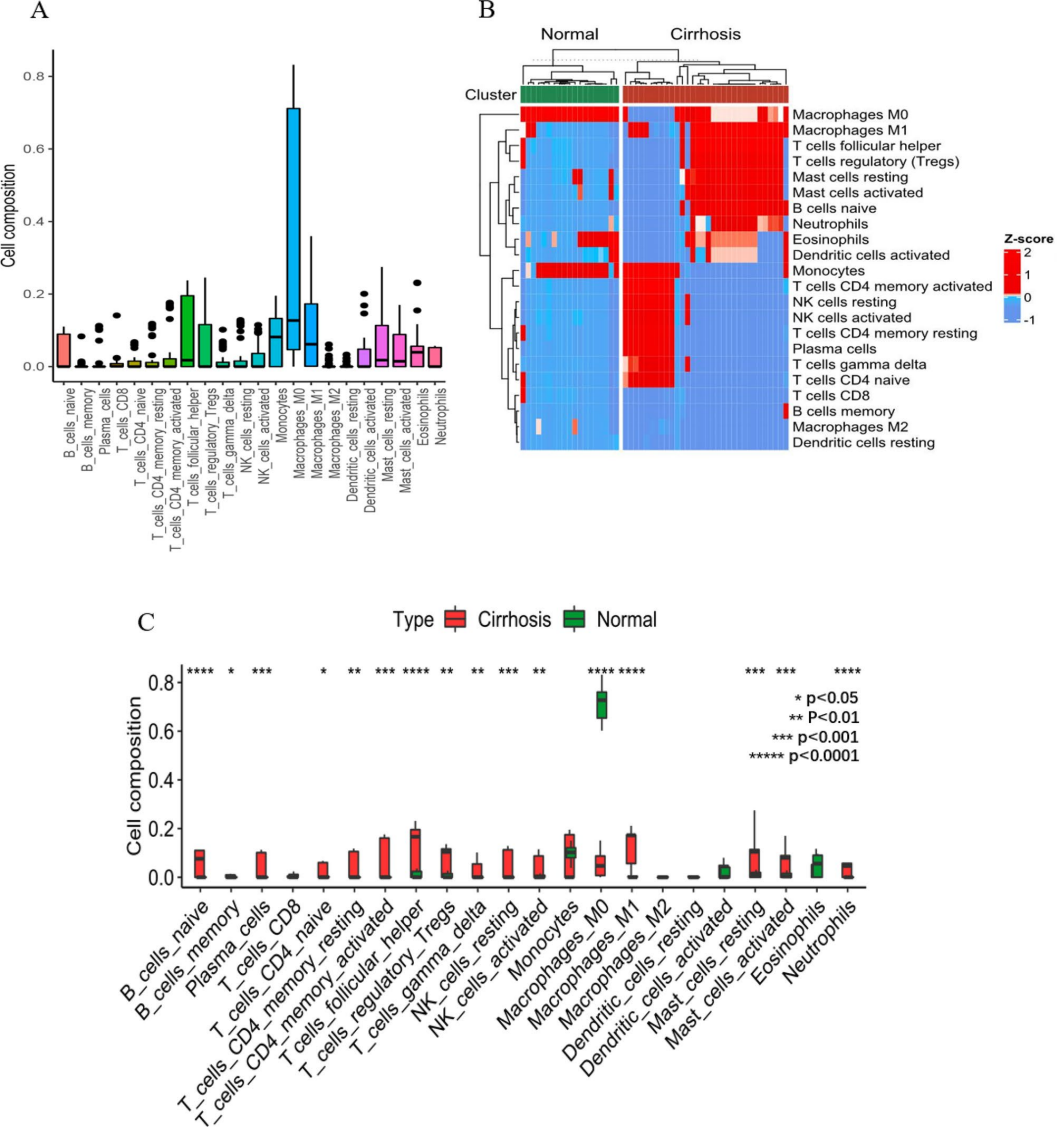
Boxplot (**A**) shows the proportion of 22 immune cells in all samples. Heatmap (**B**) shows the expression change of the 22immune cells between normal liver and cirrhosis samples from two databases. Boxplot (**C**) shows the expression levels of 22 immune cells in normal liver and cirrhosis samples. Red: cirrhosis; Blue: normal.

CIBERSORT algorithm was employed to investigate the top five immune cells (Monocytes-M0, Monocytes-M1, T-cell-CD4-memory-activated, T-cells-regulatory-Trags and T-cell-CD4-resting) expressed within all the tissues (Fig. 3A). Then percentages of the 22 immune cells subsets in healthy liver and cirrhosis tissues were displayed (Fig. 3B). Finally, the significant variance of 16 kinds of immune cells between 19 normal people and 32 cirrhosis patients was illustrated (Fig. 3C).

### Construction of PPI network and identification of hub DEGs

**Figure 4:**
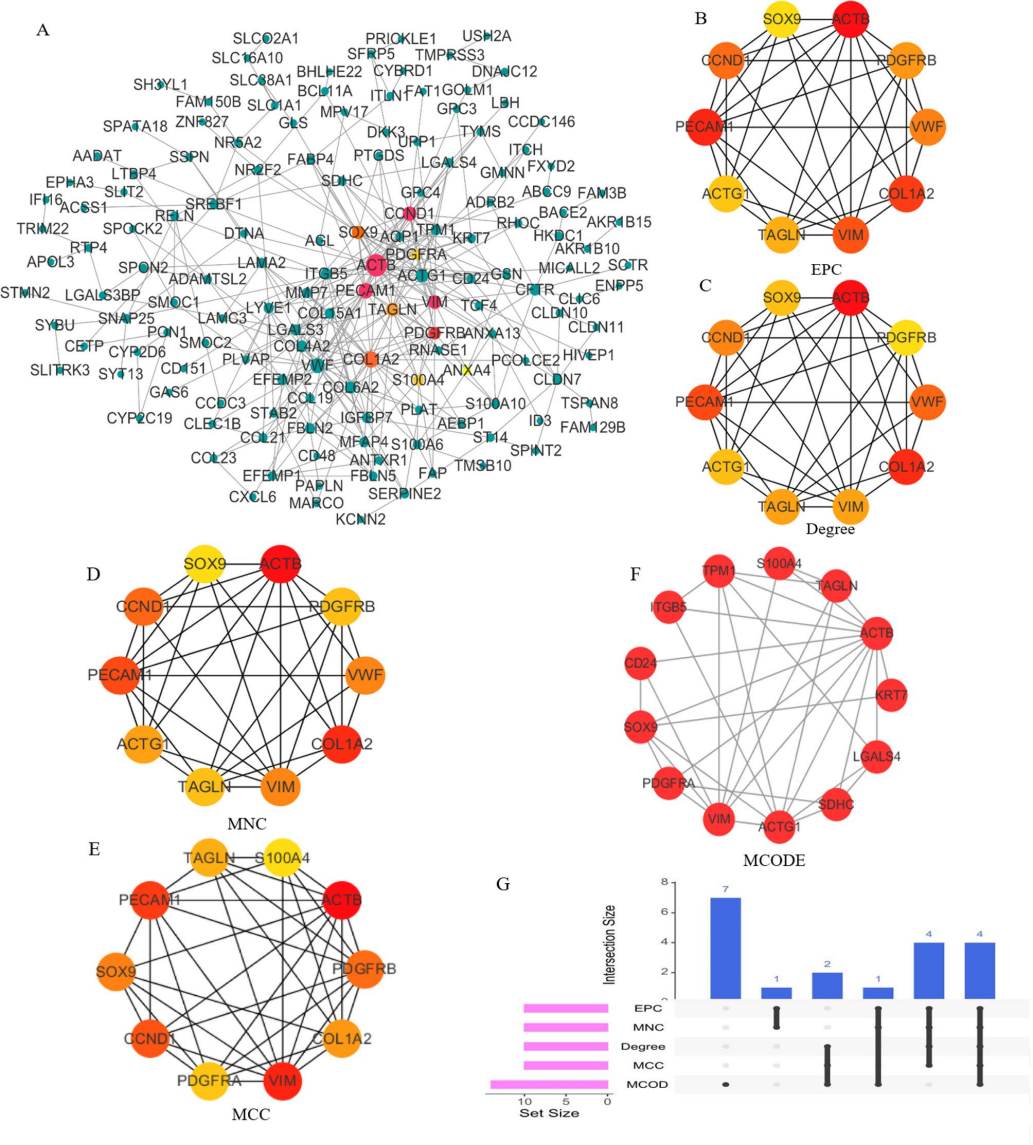
Construction of PPI network and identification of hub DEGs. (**A**) The PPI network of differentially co-expressed cirrhosis-related genes. (**B**) The top 10 hub DEGS with the highest connectivity extracted by EPC (color depth for ranking of hub DEGs). (**C**) Top 10 hub DEGs with the highest connectivity identified by Degree (color depth for ranking of hub DEGs). (**D**) Top 10 hub DEGs with the highest connectivity identified by MNC (color depth for ranking of hub DEGs). (**E**) Top 10 hub DEGs with the highest connectivity identified by MCC (color depth for ranking of hub DEGs). (**F**) Top 10 hub DEGs with the highest connectivity identified by MCODE (color depth for ranking of hub DEGs). (**G**) The UpSet diagram shows the intersection of the top 10 HUB genes obtained by the above five methods (Pink bar represents the five methods, and blue bar represents the intersecting genes).

**Figure 5:**
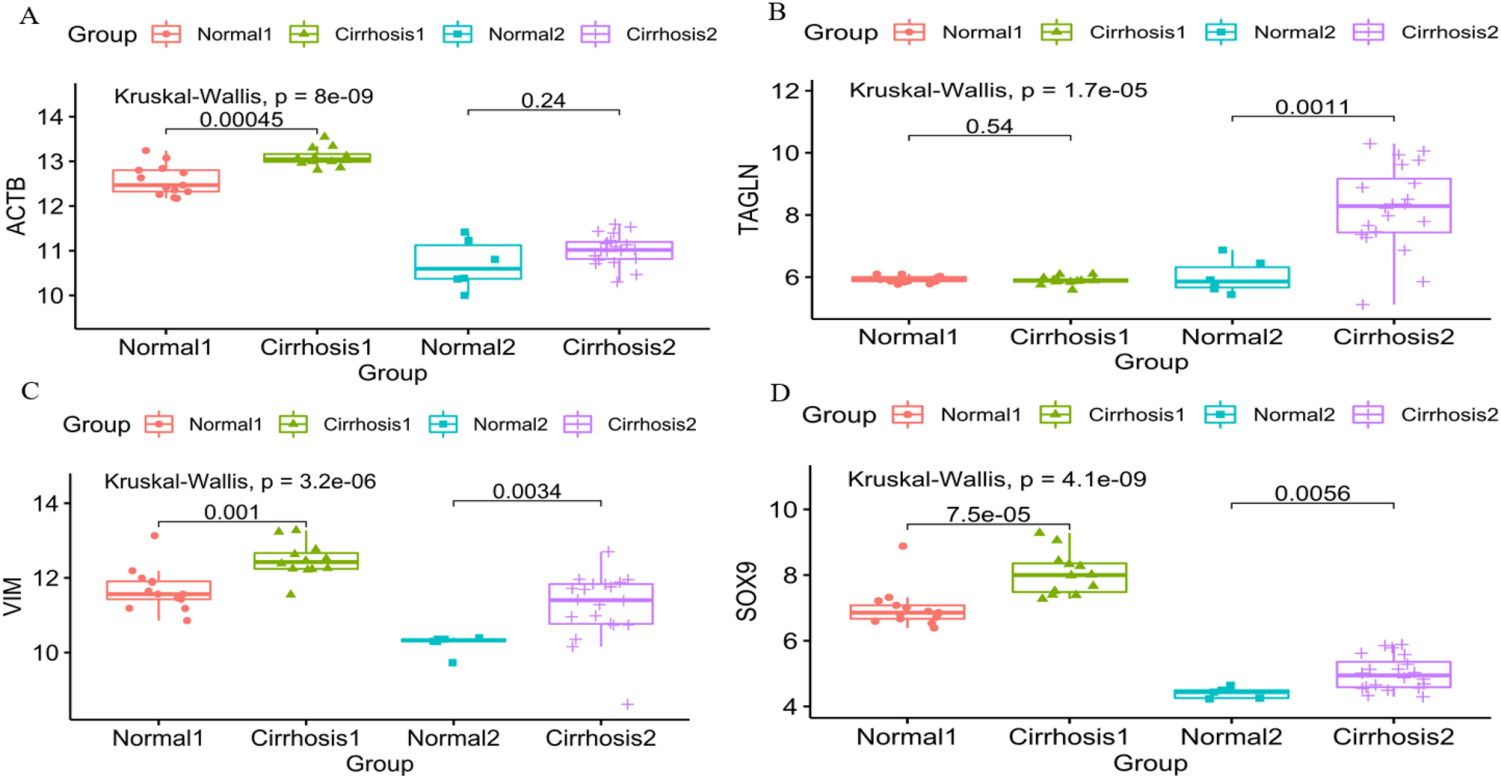
Expression analysis of hub DEGs between normal and cirrhosis samples from two datasets. (**A**) The expression pattern of *ACTB* in normal and cirrhosis samples from GSE89377 and GSE139602. (**B**) The expression pattern of *TAGLN* in normal and cirrhosis samples from GSE89377 and GSE139602. (**C**) The expression pattern of *VIM* in normal and cirrhosis samples from GSE89377 and GSE139602. (**D**) The expression pattern of *SOX9* in normal and cirrhosis samples from GSE89377 and GSE139602.

A PPI network of the cirrhosis-related co-expressed DEGs was constructed in two expression profiles (Fig. 4A). Meanwhile, DEGs with the highest connectivity were selected by five calculation methods (Fig. 4B–F). Subsequently, four hub genes (In GSE89377 and GSE139602, the Log2FC values of *ACTB*, *TAGLN*, *VIM* and *SOX9* are 0.51, 0.95, 0.78, 1.05 and 0.76, 2.20, 1.10, 2.76, respectively.) were obtained from intersection of the top ten Hub DEGs extracted by the five methods (Fig. 4G). Finally, the variance between cirrhosis and normal tissues within two databases was acquired (Fig. 5A–D).

### Diagnostic effectiveness of Hub DEGs in cirrhosis

**Figure 6:**
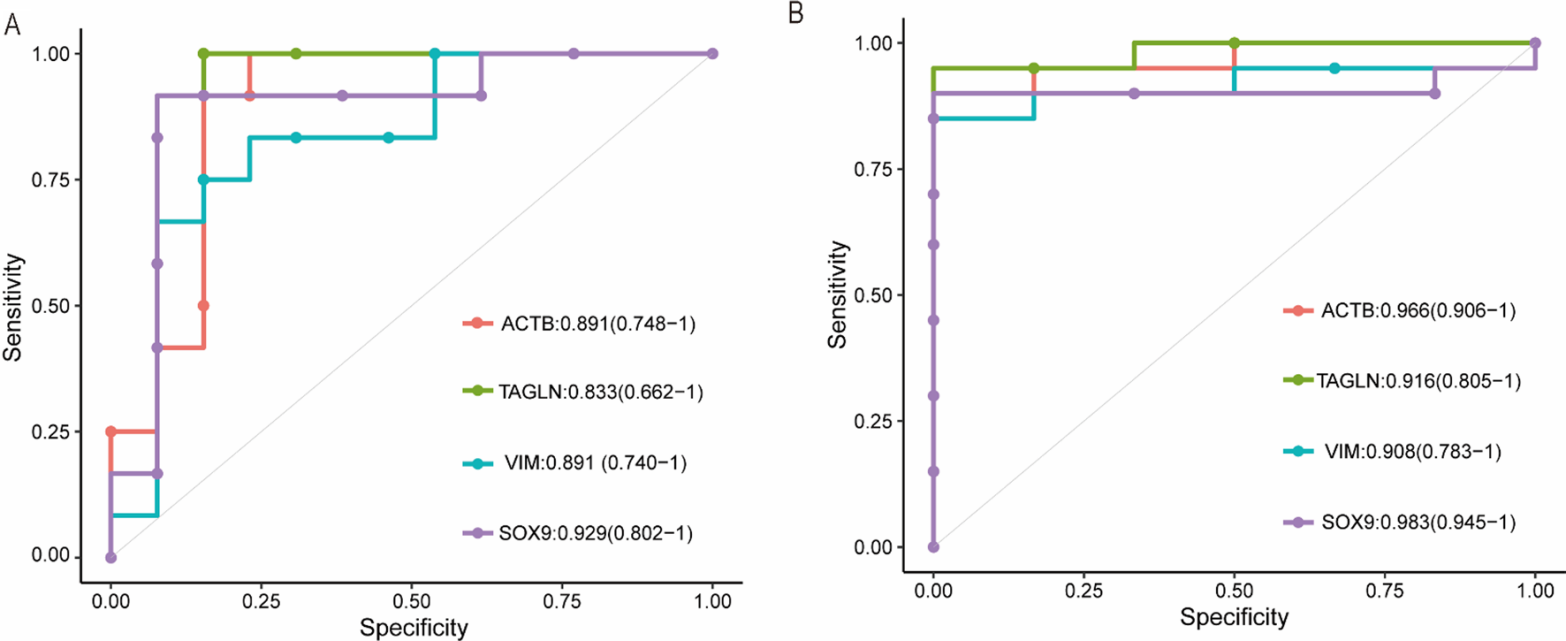
The ROC curve of diagnostic effectiveness of Hub DEGs in cirrhosis (**A**, **B**) ROC curve of *ACTB*, *TAGLN, VIM* and *SOX9* in the data of GSE89377 and GSE139602 respectively.

Our results indicated that these four Hub DEGs screened out by the five methods have a favorable diagnostic value in the two gene expression profiles, in GSE89377 dataset with an AUC of 0.89 (95% CI 0.74–1.00) in ACTB, AUC of 0.83 (95% CI 0.66–1.00) in TAGLN, and AUC of 0.89 (95% CI 0.74–1.00) in VIM, and AUC of 0.92 (95% CI 0.80–1.00) in SOX9, and in GSE139602 dataset with an AUC of 0.96 (95% CI 0.90–1.00) in ACTB, AUC of 0.91 (95% CI 0.80–1.00) in TAGLN and AUC of 0.90 (95% CI 0.78–1.00) in VIM, and AUC of 0.98 (95% CI 0.94–1.00) in SOX9 (Fig. 6A, B).

### Relationship analyses between hub DEGs and immune cells

**Figure 7:**
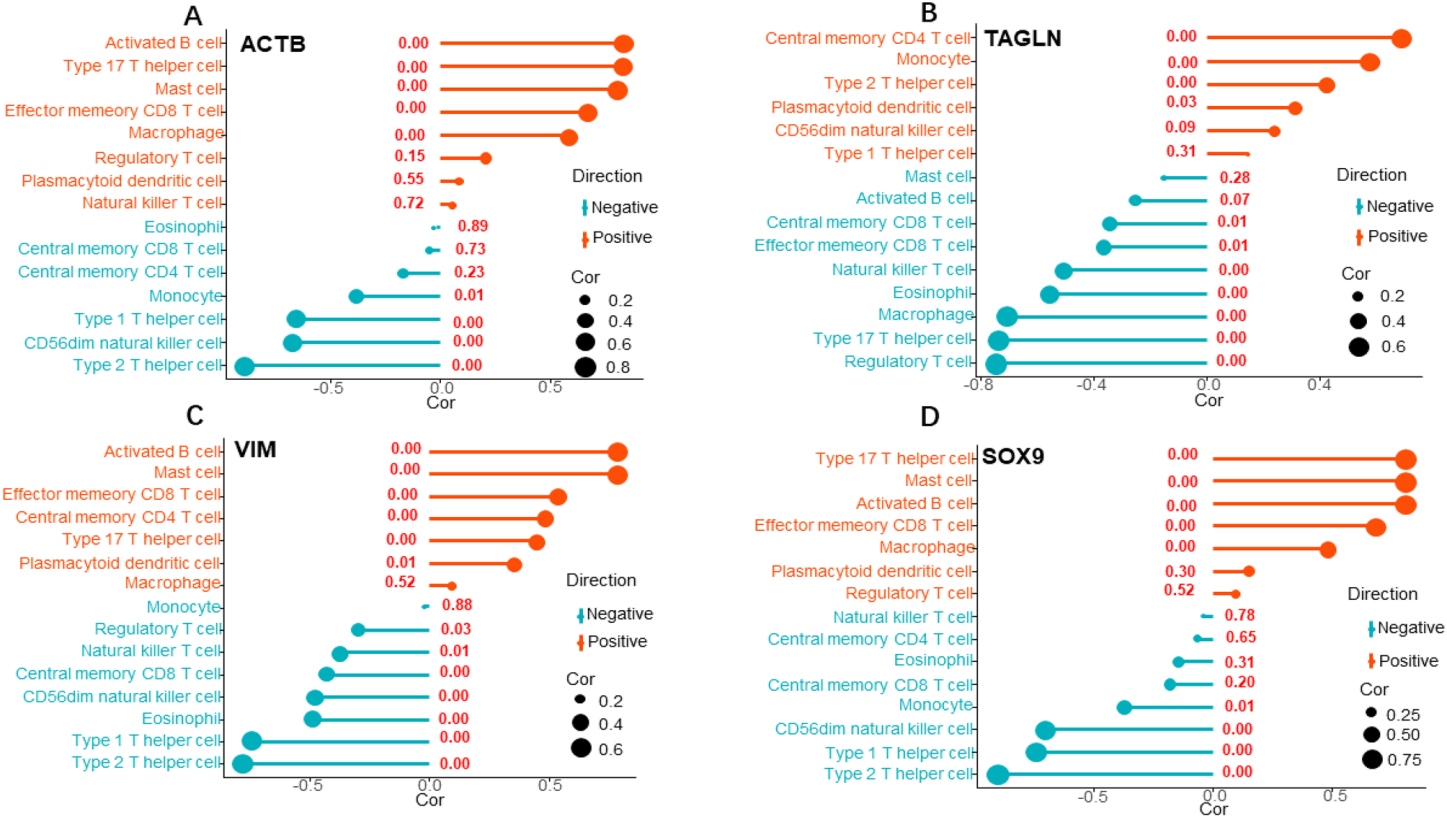
Correlations between hub DEGs and infiltrating immune cells. (**A**) Correlation between ACTB and infiltrating immune cells. (**B**) Correlation between TAGLN and infiltrating immune cells. (**C**) Correlation between VIM and infiltrating immune cells. (**D**) Correlation between SOX9 and infiltrating immune cells. Size of the dot represents the strength of the correlation between key immune related genes and immune cells; the larger (or smaller) the dot is, the stronger (or weaker) the correlation is. Color of the dot represents the negative or positive correlation; green: negative correlation, red: positive correlation. The number above the dot represents the *P* value; *P* < 0.05 and absolute value (Cor) > 0.5 are considered statistically significant.Cor:correlatio.

*ACTB* was positively related with B cell activated (r = 0.83, *P* = 5.71E−08), Type 17 T helper cell (r = 0.83, *P* = 1.51E−07), macrophage (r = 0.58, *P* = 7.16E−06), mast cell (r = 0.80, *P* = 1.51E−07), effector memory CD8 T-cell(r = 0.67, *P* = 4.62E−14), and significantly negatively correlated with CD56dim natural killer cell (r = −0.67, *P* = 5.71E−08), T helper cell of Type 1 (r = −0.66, *P* = 1.51E−07), and T helper cell of Type 2 (r = −0.89, *P* = 0) (Fig. 7A).

*TAGLN* was positively correlated with central memory CD4 T cell (r = 0.69, *P* = 2.04E−08), and monocyte (r = 0.58, *P* = 9.30E−06), and negatively correlated with eosinophil (r = −0.56, *P* = 2.25E−05), macrophage (r = −0.71, *P* = 7.49E−09), T-cell regulatory (r = −0.74, *P* = 3.95E-10), natural killer T cell (r = −0.51, *P* = 0.00), and T helper cell of Type 17 (r = −0.73, *P* = 9.22E−10) (Fig. 7B).

*VIM* was positively correlated to B cell activated (r = 0.78, *P* = 1.38E−11), Effector memory CD8 T-cell (r = 0.53, *P* = 5.69E−05), and mast cell (r = 0.78, *P* = 1.46E−11), and negatively correlated to T helper cell of Type 1 (r = -0.73, *P* = 5.78E−10), and T helper cell of Type 2 (r = −0.78, *P* = 1.78E−11) (Fig. 7C).

*SOX9* was positively correlated with B cell activated (r = 0.80, *P* = 1.10E−12), Effector memory CD8 T cell (r = 0.68, *P* = 3.94E−08), Mast cell (r = 0.81, *P* = 1.01E−12), and helper cell of Type 17 T (r = 0.81, *P* = 9.42E−13), and negatively correlated with natural killer cell CD56dim (r = −0.70, *P* < 8.02E−09), T helper cell of Type 1 (r = −0.74, *P* = 4.06E−10), and T helper cell of Type 2 (r = −0.90, *P* = 0) (Fig. 7D).

## Discussion

Cirrhosis, a most common liver disease, is triggered by alcoholic liver disease, chronic viral hepatitis, non-alcoholic fatty liver disease, non-alcoholic steatohepatitis, or other causes^25,26^. To date, despite the various treatments, including dietary control, drug therapy, and surgical intervention^27^, the treatment effect of cirrhosis remains modest, with high rates of adverse effects and risks of liver function deterioration. Therefore, screening the diagnostic and therapeutic biomarkers is urgently needed to prevent the occurrence and development of cirrhosis. Increasing evidence has suggested the association of cirrhosis with immune-inflammatory responses^4,28^. Interestingly, increasing studies have showed blocking the accumulation of extracellular matrix to inhibit inflammatory cytokines might be a promising therapy for cirrhosis^4,28,29^. In this study, we identified immune relevant genes and explored the effect of immune cell infiltration in cirrhosis using bioinformatics analysis.

Chronic liver injury is implicated in chronic liver cell and epithelial/endothelial barrier damage, as well as the inflammatory cytokine releasement and hepatic myofibroblasts activation, eventually leading to overproduction of extracellular matrix and scar formation^28^. Among the above factors, activated hepatic stellate cells (HSCs), the major cellular source of matrix-producing myofibroblasts, play a significant role in the initiation and progression of liver fibrosis. Paracrine signals from resident and inflammatory cells (such as hepatocytes, hepatic macrophages, natural killer/natural killer T cells and platelets) could directly or indirectly regulate HSC differentiation and activation^30^. In our study, a total of 299 DEGs were screened out as candidate biomarkers. We identified the underlying mechanism of DEGs by enrichment function analysis. GO enrichment exploration indicated that DEGs were markedly correlated with astrocyte differentiation, dendritic cell apoptotic process, establishment of lymphocyte polarity immunological synapse formation, negative regulation of macrophage derived foam cell differentiation, extracellular matrix and collagen trimer and microfibril in external matrix structural constituent. Further, these genes were found involved in viral protein interaction with cytokine and cytokine receptor, cell adhesion molecules, phagosome, hepatitis C, leukocyte transendothelial migration, complement and coagulation cascades pathways according to the KEGG analysis. In recent years, it is well documented that viral protein interaction with cytokine and cytokine receptor, transendothelial migration, hepatitis C, cell adhesion and complement and coagulation cascades pathways are involved in the Liver disease, including, but not limited to, liver fibrosis, hepatic encephalopathy, Hepatocellular cholangiocarcinoma^31–34^. In conclusion, it is proved that the enrichment pathway of our studied genes is basically consistent with the current pathological mechanism of liver cirrhosis.

Accumulating studies, believe that neutrophils, macrophages, NK cells and CD4T cells are involved in the potential proinflammatory and profibrotic immune mechanisms in the process of cirrhosis. Neutrophils, which are usually recruited to the liver at the early stage of liver injury to clear apoptotic hepatocytes^35^, can release cell-free DNA with a strong pro-inflammatory effect^36^. Cirrhosis mouse model showed alleviated liver fibrosis after deletion or ablation of neutrophil chemokines^37,38^. In the fibrosing process, inflammation and the macrophages in the liver, activate HSCs by producing cytokines and chemokines^39^. Macrophages also promote myofibroblast apoptosis by expressing MMP9 and TRAIL^40^, and thus enhance epithelial-mesenchymal transformation (ECM) degradation to alleviate fibrosis in rodent models^40,41^. Activated liver-associated NK cells may be antifibrogenic by killing HSCs and releasing IFNγ^42^. However, CD4 + T lymphocytes inhibit NK cells through interacting with NK cells and activating hematopoietic stem cells, which is conducive to hematopoietic stem cells^43^. Using CIBERSORT algorithm to analyze the immune differences between normal and cirrhotic tissues, we found increased infiltration of neutrophils, regulatory T cells, T cells follicular helper, gamma delta T cells, CD4 T memory activated cells, CD4 T memory resting cells, CD4 T memory naive cells, resting NK cells, activated NK cells, activated mast cells, resting mast cells, naïve B cells, memory B cells, plasma cells, and M1 macrophages, as well as reduced infiltration of M0 macrophages may be associated with cirrhosis pathogenesis. consistent with previous studies. These results indicate that the immune cells involved in the immune-inflammatory process of liver cirrhosis is extremely complex, and we speculate that liver fibrosis may be the result of the imbalance between anti-fibrosis and fibrosis.

Based on the results of GO, KEGG and immune analysis, four potential hub DEGs (*ACTB, TAGLN, VIM* and *SOX9*) were identified as the core genes for the immune-inflammatory responses. *ACTB* (beta-actin), a constitutive housekeeping gene^44^ and highly conserved cytoskeleton protein, is generally dispersed in all eukaryotic cells and plays vital roles in cell division, cell migration, immune response and gene expression^45–47^. The 3'-UTR of *ACTB* is closely correlated with the development of liver cancer^48^. Although the correlation between *ACTB* level and cirrhosis has not been evidenced, a recent study has shown that *ACTB* is involved in circulatory inflammation and angiogenesis^49^. Our study found that *ACTB* was positively related with macrophage, mast cell, B cell activated, Type 17 T helper cell, effector memory CD8 T-cell. Therefore, we hypothesized that *ACTB* gene may be involved in the immune response related to liver fibrosis.

*TAGLN,* an actin crosslinking protein expressed in fibroblasts, endothelial cells, and immune cells, interacts with calcium to regulate cytoplasm contraction^50,51^. The overexpression of *TAGLN* protein has been observed in patients with human hepatocellular carcinoma^52^. In addition, in the mouse model, vascular endothelial growth factor A can simultaneously activate *TAGLN* promoter and elongate endothelial cells, and *TAGLN* is speculated as a regulatory factor of angiogenesis^53–55^. In present study, *TAGLN* was positively correlated with monocyte, central memory CD4 T cell, but negatively correlated with macrophage. Hence, Whether *TAGLN* participate in cirrhosis via activating monocyte and CD4 T cell to achieve matrix remodeling and migration, as well as cell differentiation and invasion needs further investigation.

*VIM* (vimentin), a member of the intermediate filamentous family, is specifically found in connective tissues^56^. The *VIM* gene encodes vimentin, not only preserving cell morphology and stabilizing cytoskeleton interactions, but also playing a significant role in cell migration, inflammation, signal transduction and other biological processes^57^. Recently, *VIM,* as one of *SOX9* targets, has been evidenced to adjust the advance of liver fibrosis^58^, which is in line with the findings of the present study. In our study, the expression of *VIM* was positively correlated to B cell activated Effector memory CD8 T-cell and mast cell. Thus, we reveal that the *VIM* gene may play an important role in the immune response to liver fibrosis through o B cell activated Effector memory CD8 T-cell and mast cells.

Previous, researches have shown that the ectopic expression of gender-determining transcription factor Y-box 9 (*SOX9*) takes charge of type 1 collagen production in activated hematopoietic stem cells^59^. A clinical biopsy study of cirrhosis has found that the *SOX9* expression levels in chronic liver disease is related to the severity of fibrosis, and thus can precisely predict cirrhosis progression^60^. Another study has identified that extracellular protein epimorphin regulates the excessive ECM environment generated by activated HSCs, by down-regulating pro-fibrotic *SOX9*^61^. Recently, a molecular study proved that HBV activates *SOX9* expression via increasing *SOX9* promoter activity. Interestingly, in turn, *SOX9* inhibits HBV replication by straightly binding to EnhII/Cp to deactivate EnhII/Cp^62^. The above findings all support *SOX9* as a hub regulator of fibrotic ECM in the progression of liver fibrosis^63,64^, which is also consistent with our present study. *SOX9* was positively correlated with B cell activated, Effector memory CD8 T cell, Mast cell and helper cell of Type 17 T in present study. These data indicate that *SOX9* plays a key role in hepatic fibrosis progression and thus is useful as an immunotherapeutic target.

Our further investigation into the association between these DEGs and immune infiltration also revealed the correlations between *ACTB, TAGLN, VIM, SOX9* and immune cells, supporting that these genes play a vital role in cirrhosis via regulating immune infiltration.

## Conclusion

In summary, we found *ACTB, TAGLN, VIM* and *SOX9* are the potential key biomarkers of liver cirrhosis. Moreover, the correlation between these four hub DEGs and immune cells may play a critical role in the pathogenesis of cirrhosis.

## Limitation

Several limitations should be highlighted in our study. First, our findings are produced by a microarray and immune-related analysis based on gene expression and immunological databases. Second, although the results are enlightening, how these cirrhosis-related key DEGs and immune cells contribute to cirrhosis remains unknown. Therefore, further experiments are needed to verify the biological function of these genes.

## Supplementary Information

Supplementary Information 1.

Supplementary Information 2.

Supplementary Information 3.

Supplementary Information 4.

## Data Availability

All the data were acquired from the Gene Expression Omnibus (GEO) database. GSE89377_series_matrix.xls : The GSE89377 series based on the GPL16947 platform (Illumina HumanHT-12 V3.0 expression biochip). (https://www.ncbi.nlm.nih.gov/geo/query/acc.cgi). GSE139602_series_matrix.xls: The GSE139602 series based on the GPL13667 platform ([HG-U219] Affymetrix Human Genome U219 array (https://www.ncbi.nlm.nih.gov/geo/query/acc.cgi). LM22.xls: The LM22 gene file contained 22 types immune cells and 547 genes was used to define the 22 immune cell subcategories and analyze cirrhosis data, which were attained from the CIBERSORT web portal, Supplementary Table1 in Supplementary information section. (https://www.nature.com/articles/nmeth.3337#MOESM207).

## Abbreviations

DEGs: Differentially expressed genes
GO: Gene ontology
KEGG: Kyoto Encyclopedia of Genes and Genomes
PPI: Protein–protein interaction
