# Supplementary material for: Identification of key genes related to immune infiltration in cirrhosis via bioinformatics analysis
Source: Sci Rep. 2023 Feb 1;13:1876. doi: 10.1038/s41598-022-26794-8 (PMC9892033; doi:10.1038/s41598-022-26794-8)
Supplement: Supplementary file 1 — Supplementary Information 1. [file 41598_2022_26794_MOESM1_ESM.docx]

Description in brief for the data provided in GSE89377_series_matrix.xls, GSE139602_series_matrix.xls, LM22.xls, GSE139602_DEGs.csv and GSE139602_DEGs.csv,

GSE89377_series_matrix.xls ：The GSE89377 series contained 13 normal human liver tissue specimens and 12 human cirrhosis tissue specimens based on the GPL16947 platform (Illumina HumanHT-12 V3.0 expression biochip).

https://www.ncbi.nlm.nih.gov/geo/query/acc.cgi

GSE139602_series_matrix.xls：The GSE139602 series contained 6 normal human liver tissue specimens and 20 human cirrhosis tissue specimens based on the GPL13667 platform ([HG-U219] Affymetrix Human Genome U219 array).

https://www.ncbi.nlm.nih.gov/geo/query/acc.cgi

LM22.xls: The LM22 gene file contained 22 types immune cells and 547 genes was used to define the 22 immune cell subcategories and analyze cirrhosis data, which were attained from the CIBERSORT web portal, Supplementary Table1 in Supplementary information section. (<https://www.nature.com/articles/nmeth.3337#MOESM207>).

GSE89377_DEGs.csv: The GSE89377_DEGs file contained a full length Differential gene expression (DEGs) table with probe id, logFC, pValue, adj.P.Val, t value, and Gene.

GSE139602 DEGs.csv:The GSE139602_DEGs file contained a full length Differential gene expression (DEGs) table with probe id, logFC, pValue, adj.P.Val, t value, and Gene.

GO.csv: The GO file contained GO enrichment analysis along with Gene counts and Gene names.

KEGG.csv: The KEGG file contained Pathway enrichment analysis along with Gene counts and Gene names.

PPI_topology_Table.csv: The PPI_topology_Table file contained PPI topology Table with node degree, betweenness centrality, stress centrality, and closeness centrality.
