# Supplementary material for: Identification of key genes related to immune infiltration in cirrhosis via bioinformatics analysis
Source: Sci Rep. 2023 Feb 1;13:1876. doi: 10.1038/s41598-022-26794-8 (PMC9892033; doi:10.1038/s41598-022-26794-8)
Supplement: Supplementary file 2 — Supplementary Information 2. [file 41598_2022_26794_MOESM2_ESM.docx]

Addition file: Table 1
Clinical data information of GSE89377 and GSE139602
Dataset	Country	Sample ID	title	Diagnosis	
GEO: GSE89377	South Korea	GSM2367554	N-01	Normal	
GEO: GSE89377	South Korea	GSM2367555	N-02	Normal	
GEO: GSE89377	South Korea	GSM2367556	N-03	Normal	
GEO: GSE89377	South Korea	GSM2367557	N-04	Normal	
GEO: GSE89377	South Korea	GSM2367558	N-05	Normal	
GEO: GSE89377	South Korea	GSM2367559	N-06	Normal	
GEO: GSE89377	South Korea	GSM2367560	N-07	Normal	
GEO: GSE89377	South Korea	GSM2367561	N-08	Normal	
GEO: GSE89377	South Korea	GSM2367562	N-09	Normal	
GEO: GSE89377	South Korea	GSM2367563	N-10	Normal	
GEO: GSE89377	South Korea	GSM2367564	N-11	Normal	
GEO: GSE89377	South Korea	GSM2367565	N-12	Normal	
GEO: GSE89377	South Korea	GSM2367566	N-13	Normal	
GEO: GSE89377	South Korea	GSM2367587	CS-01	Cirrhosis	
GEO: GSE89377	South Korea	GSM2367588	CS-02	Cirrhosis	
GEO: GSE89377	South Korea	GSM2367589	CS-03	Cirrhosis	
GEO: GSE89377	South Korea	GSM2367590	CS-04	Cirrhosis	
GEO: GSE89377	South Korea	GSM2367591	CS-05	Cirrhosis	
GEO: GSE89377	South Korea	GSM2367592	CS-06	Cirrhosis	
GEO: GSE89377	South Korea	GSM2367593	CS-07	Cirrhosis	
GEO: GSE89377	South Korea	GSM2367594	CS-08	Cirrhosis	
GEO: GSE89377	South Korea	GSM2367595	CS-09	Cirrhosis	
GEO: GSE89377	South Korea	GSM2367596	CS-10	Cirrhosis	
GEO: GSE89377	South Korea	GSM2367597	CS-11	Cirrhosis	
GEO: GSE89377	South Korea	GSM2367598	CS-12	Cirrhosis	
GEO: GSE139602	Spain	GSM4144550	Healthy Liver biopsy(LMS1)	Healthy	
GEO: GSE139602	Spain	GSM4144551	Healthy Liver biopsy(LMS2)	Healthy	
GEO: GSE139602	Spain	GSM4144552	Healthy Liver biopsy(LMS3)	Healthy	
GEO: GSE139602	Spain	GSM4144553	Healthy Liver biopsy(LMS4)	Healthy	
GEO: GSE139602	Spain	GSM4144554	Healthy Liver biopsy(LMS5)	Healthy	
GEO: GSE139602	Spain	GSM4144555	Healthy Liver biopsy(LMS6)	Healthy	
GEO: GSE139602	Spain	GSM4144561	Compensasted Cirrhosis Liver biopsy(LM022)	Compensasted Cirrhosis 	
GEO: GSE139602	Spain	GSM4144562	Compensasted Cirrhosis Liver biopsy(LM027)	Compensasted Cirrhosis 	
GEO: GSE139602	Spain	GSM4144563	Compensasted Cirrhosis Liver biopsy(LM029)	Compensasted Cirrhosis 	
GEO: GSE139602	Spain	GSM4144564	Compensasted Cirrhosis Liver biopsy(LM030)	Compensasted Cirrhosis 	
GEO: GSE139602	Spain	GSM4144565	Compensasted Cirrhosis Liver biopsy(LM047)	Compensasted Cirrhosis 	
GEO: GSE139602	Spain	GSM4144566	Compensasted Cirrhosis Liver biopsy(LM051)	Compensasted Cirrhosis 	
GEO: GSE139602	Spain	GSM4144567	Compensasted Cirrhosis Liver biopsy(LM144)	Compensasted Cirrhosis 	
GEO: GSE139602	Spain	GSM4144568	Compensasted Cirrhosis Liver biopsy(LM057)	Compensasted Cirrhosis 	
GEO: GSE139602	Spain	GSM4144569	Compensasted Cirrhosis Liver biopsy(LM050)	Decompensasted Cirrhosis 	
GEO: GSE139602	Spain	GSM4144570	Compensasted Cirrhosis Liver biopsy(LM002)	Decompensasted Cirrhosis 	
GEO: GSE139602	Spain	GSM4144571	Compensasted Cirrhosis Liver biopsy(LM012)	Decompensasted Cirrhosis 	
GEO: GSE139602	Spain	GSM4144572	Compensasted Cirrhosis Liver biopsy(LM018)	Decompensasted Cirrhosis 	
GEO: GSE139602	Spain	GSM4144573	Compensasted Cirrhosis Liver biopsy(LM036)	Decompensasted Cirrhosis 	
GEO: GSE139602	Spain	GSM4144574	Compensasted Cirrhosis Liver biopsy(LM077)	Decompensasted Cirrhosis 	
GEO: GSE139602	Spain	GSM4144575	Compensasted Cirrhosis Liver biopsy(LM009)	Decompensasted Cirrhosis 	
GEO: GSE139602	Spain	GSM4144576	Compensasted Cirrhosis Liver biopsy(LM023)	Decompensasted Cirrhosis 	
GEO: GSE139602	Spain	GSM4144577	Compensasted Cirrhosis Liver biopsy(LM054)	Decompensasted Cirrhosis 	
GEO: GSE139602	Spain	GSM4144578	Compensasted Cirrhosis Liver biopsy(LM013)	Decompensasted Cirrhosis 	
GEO: GSE139602	Spain	GSM4144579	Compensasted Cirrhosis Liver biopsy(LM005)	Decompensasted Cirrhosis 	
GEO: GSE139602	Spain	GSM4144580	Compensasted Cirrhosis Liver biopsy(LM035)	Decompensasted Cirrhosis 	
