# Supplementary material for: Identification of key genes related to immune infiltration in cirrhosis via bioinformatics analysis
Source: Sci Rep. 2023 Feb 1;13:1876. doi: 10.1038/s41598-022-26794-8 (PMC9892033; doi:10.1038/s41598-022-26794-8)
Supplement: Supplementary file 3 — Supplementary Information 3. [file 41598_2022_26794_MOESM3_ESM.docx]

Addition file: Table 2 
Differentially expressed genes (DEGs) name
RORA	SLITRK3	NUBPL	VMO1	ACSL5	NCOR1	AMHR2	NAMPT	HSD17B14	PEG3	KCNAB1	MT1B	EFEMP1	
SGK3	PALM2	UBR3	HIVEP1	WEE1	PPARGC1A	NFKBIA	SUCNR1	MT1H	IGFALS	CLEC1B	TPST1	VWF	
PSME4	FCN2	GCNT2	SDHC	GFOD2	AOX1	MARCO	TTC36	AADAT	THOP1	ANKRD35	RABEPK	FBLN5	
NR5A2	ZCCHC6	KBTBD11	LRRFIP2	DNAJC12	SLC1A1	SLC13A5	GCAT	HEY2	CACNA1H	SOCS2	USH2A	COL4A2	
ITCH	PPP3R1	DMD	CLEC4G	KCNN2	SLC16A10	TSPAN7	IRS2	COBLL1	CNDP1	MAN1C1	ERGIC3	TMSB10	
KLHL15	CLEC4M	RALGPS2	PACSIN3	LONP2	JUND	FNIP2	MAT1A	PON1	AGL	MBNL2	GSN	GEM	
CETP	STYX	LYVE1	CNST	DNASE1L3	AVPR1A	ZYG11B	MT1E	FAM151A	SMOC1	TAT	AKR1B10	VTCN1	
GPC6	OSBPL8	FAM46A	CFL2	UPP1	TRIB1	ABCC9	FCN3	IL1RN	SGMS2	FOLH1	C7	SMOC2	
RBMS1	STRN3	RAD21	HPR	STAB2	GALK1	RCAN1	RNF125	PCDH20	CCL3	ST3GAL6	LGALS3BP	CCND1	
CCL23	IFT88	EGFLAM	DNAJB9	MT1M	CYP2C19	DCAF11	GADD45G	SLC39A14	GCH1	SYBU	ANXA4	TAGLN	
ZRANB1	FITM1	C5	SLC38A2	ABCA1	ADRB2	MT1X	PCOLCE2	EGFR	GFRA1	CFHR3	FAM3B	PTGDS	
MBNL3	FAM134B	DEXI	RNF19A	MT1F	RELN	SAMD4A	LILRB5	ITLN1	OIT3	GLUD1	SLCO2A1	LUC7L3	
GMNN	CD151	CLDN10	TBC1D10C	FXYD2	CCDC3	CFTR	SCTR	BACE2	PRICKLE1	CCL19	SPOCK2	LGALS4	
STMN2	VSIG2	RNASE1	LAMA2	ACTB	SPINT2	AEBP1	SEL1L3	FBLN2	S100A4	SCRN1	NKD2	LDOC1	
EPHA3	GPC3	RTP4	CD24	PDGFRA	SSPN	CYBRD1	DMKN	COL6A2	CLDN7	CCDC146	CYP2D6	PLVAP	
SH3YL1	ZBED5	ST14	LBH	S100A10	MOXD1	ENPP5	TMPRSS3	CDH6	SYT13	SUSD2	MMP7	FABP4	
MICALL2	SDCBP2	LRRC1	APOL3	TRIM22	ADAMTSL2	COL15A1	VIM	TESC	IGSF3	KRT7	GPC4	BHLHE22	
BCL11A	AKR1B15	AQP1	SELM	GLS	WDR13	CLIC6	LAMC3	ZNF827	CHST9	C1orf106	SLIT2	DCDC2	
GOLM1	ID3	SLC38A1	KIAA1522	DEFB1	SOX9	LGALS3	FAP	PECAM1	IFI16	S100A6	GAS6	IGFBP7	
CLDN11	APBB3	FAM129B	SFRP5	RCAN2	PLCXD3	SPON2	LTBP4	PDGFRB	FAT1	CXCL6	HKDC1	TSHZ2	
PAPLN	TSPAN8	ANXA13	FAM150B	DTNA	KRT23	C1orf198	SLFN11	ZMAT3	CITED4	TMEM200A	CRIP1	ACSS1	
CD48	SPATA18	DKK3	CCL21	PLAT	MPV17	RHOC	ANTXR1	TYMS	SREBF1	COL1A2	SERPINE2	C15orf52	
ACTG1	ZNF83	NR2F2	HLA-A	ATP8B2	SNRPN	MFAP4	SNAP25	TPM1	ITGB5	CSNK1G2	EFEMP2	TCF4	
